# The Usability and Feasibility of a Dietary Intake Self-Monitoring Application in a Population with Varying Literacy Levels

**DOI:** 10.3390/jpm14091001

**Published:** 2024-09-20

**Authors:** Beenish Moalla Chaudhry, Katie A. Siek, Kay Connelly

**Affiliations:** 1 School of Computing and Informatics, University of Louisiana at Lafayette, Lafayette, LA 70504, USA; 2 Luddy School of Informatics, Computing and Engineering, Indiana University, Bloomington, IN 47408, USA; ksiek@iu.edu; 3 Office of Research and Innovation, Michigan State University (MSU), East Lansing, MI 48824, USA; conne166@msu.edu

**Keywords:** mobile application, literacy, portion size, behavior change, nutrition monitoring, chronic disease, health, diet

## Abstract

Objectives: Our aim was to study how hemodialysis patients with varying levels of literacy would use a diet and fluid intake monitoring mobile application (DIMA-P) and what would be its impact on their dietary behaviors. Materials and Methods: We developed a mobile application using user-centered methods and informed by the Integrated Theory of Health Behavior Change (ITHBC). Eight hemodialysis patients were recruited to use the application to record and monitor their diet and fluid intakes for a 6-week study. Overall, the participants had low literacy, numeracy, and technical skills. We collected the data on application usage and administered usability and context-of-use questionnaires to gain insights into the participants’ interaction with the application. The participants’ portion estimation skills and dietary self-regulation self-efficacy were assessed using various tests. In addition, interdialytic weight gain data were collected to assess the impact of app usage on the participants’ health outcomes. Results: The application usage patterns varied, with a general trend towards frequent use (*n* = 5) correlating with engagement in self-monitoring. The participants gave high comprehensibility, user-friendliness, satisfaction, and usefulness ratings, suggesting that the app was well designed and the target users could easily navigate and interact with the features. While the participants improved in estimating portion sizes, the impact on measuring skills was variable. There was also an improvement in the participants’ dietary self-regulation self-efficacy post-study. The interdialytic weight gain trends indicated a slight improvement in fluid and diet management. Conclusion: People with different literacy skills can effectively use icon-based interfaces for portion size estimation and develop personalized usage patterns to self-regulate their fluid and dietary intakes. Moreover, they can experience an enhancement in their dietary self-efficacy skills by using a mobile application aimed at providing nutritional feedback. Furthermore, this research shows that the constructs of the ITHBC are effective in promoting dietary behavior change in a population with varying literacy skills. The target users can benefit from explicitly visualizing the relationship between their health outcomes and the factors influencing those outcomes. These user ambitions could be supported by developing machine learning models. Future research should also focus on enhancing the mechanisms by which technology can further enhance each component of the ITHBC framework.

## 1. Introduction

People undergoing hemodialysis must maintain a strict diet to minimize the fluid and waste buildup in their bloodstreams between dialysis sessions. Typically, patients should not consume more than 1 L of fluid, 2 g of sodium, 2 g of potassium, and 1 g of phosphorus each day [1]. Additionally, they should aim for at least 1.2 g of protein and 35 kilocalories of energy intake per kilogram of body weight daily to sustain healthy body functions. A failure to comply with this dietary regimen can disrupt the blood’s electrolyte balance, leading to adverse health outcomes, such as muscle weakness, high blood pressure, and heart failure, potentially resulting in sudden death [1].

Research indicates that most hemodialysis patients find it challenging to adhere to their fluid and dietary restrictions. According to a recent systematic review, the estimated worldwide noncompliance with fluid and diet restrictions was 60.6% (95% CI: 50–70.7) and 60.2% (95% CI: 47.3–72.5), respectively [2]. Compliance requires literacy- and numeracy-based skills like the accurate estimation, tracking, and monitoring of portion intake. Unfortunately, many hemodialysis patients are often low-literate and low-numerate [3,4], lack knowledge of food’s nutritional content, and struggle with calculating the nutrient amounts from various portion sizes [5]. Low literacy also makes it difficult for people to obtain nutritional insights from items like food packaging, nutritional labels, advertisements, plate sizes, and unit sizes, causing them to consume inappropriate amounts of nutrients, which have the potential to raise healthcare costs and increase physical health risks [6].

Dietary self-monitoring research often highlights the adoption of mobile apps for diet tracking across diverse groups, such as those with and without diabetes [7], pregnant women with a low socioeconomic status [8], and individuals grappling with eating disorders [9]. Such studies underscore the potential of mobile apps as valuable tools for diet management and introspection across various health contexts. In particular, research suggests that diet monitoring mobile applications can help chronically ill people monitor their diet quality [10], understand their dietary behaviors [11], and be encouraged to eat healthily [12]. However, mobile health adoption rates among chronic kidney disease (CKD) patients are low compared to other chronic conditions and are determined by patients’ health literacy scores [13]. Moreover, CKD patients are often older adults from low socioeconomic backgrounds—a demographic frequently overlooked by designers because age and social standing pose additional challenges to designers, including the communication and empathy difficulties related to motor, cognitive, or social skills [14]. Additionally, existing diet monitoring applications are often inaccessible to low-literacy individuals due to their text-based interfaces and lack of portion size estimation tools. Photo-based diet management applications can help, but they require waiting for feedback and may be costly [15,16]. For example, with Chung et al.’s [17] Foodprint app, users must review their food photos and notes during consultations with dietitians at a later date.

We developed an Android-based diet monitoring application, DIMA-P, to empower hemodialysis patients with limited literacy skills to estimate and track their portion intake. The development of the mobile health app followed a user-centered design process, which involved multiple iterations and refinements based on feedback from health professionals and target users [18,19,20,21,22,23,24]. This iterative process included four major revisions and three beta tests, each contributing to the enhancement of the app’s functionality and user experience. The system itself was designed with a robust database that housed nutritional information sourced from the USFDA, enabling real-time feedback on users’ dietary intake. The user interface was carefully crafted to align with the mental models of the target population, particularly those with varying levels of literacy and technical skills. Key features such as a portion size estimation module, real-time nutritional feedback, and a linear navigation style were all developed to ensure that the app was both functional and accessible.

We deployed the completed DIMA-P application in a six-week in situ pilot study, where a group of hemodialysis patients used the app to track and self-monitor their diet. We have presented an evaluation of the portion size estimation interface of the DIMA-P in a previous publication [25]. In this paper, we present the usability of the entire DIMA-P app using standardized scales, study how it was used, understand the health behavior changes instigated by the app, and investigate the impact of precise nutritional feedback on the interdialytic weight gain (IDWG) of the target users. IDWG is an important metric in this population because research shows that a low IDWG is indicative of subpar nutrition, while a high IDWG (often resulting from excessive sodium and potassium intakes) correlates with elevated blood pressure and a heightening mortality risk [26]. Conversely, an IDWG within the recommended range can signal improved nutritional health.

Usability evaluation is crucial for mobile health apps as it directly impacts their effectiveness, user engagement, and overall success [27,28]. In health interventions, especially those targeting vulnerable or chronically ill populations, usability determines whether an app will be adopted and consistently used. A well-designed, user-friendly app enhances satisfaction, reduces errors, and ensures that users can easily navigate and interact with features to achieve their health goals.

Mobile health apps often require users to self-monitor, track metrics, and make informed decisions based on feedback. Usability issues can hinder these processes, leading to non-compliance or even harm. Evaluating usability early ensures the app is intuitive, accessible, and aligned with users’ needs, fostering acceptance and sustained use, which are key to behavioral change. Given the scarcity of mobile apps for managing chronic conditions with strict dietary regimens [13], developing and evaluating user-centric tools like the DIMA-P for hemodialysis patients by determining its usability and feasibility can set a precedent for broader adoption and innovation in mHealth solutions [29]. Based on these reasons, the primary research questions for this study are as follows:

RQ1: How would the participants use the DIMA-P to track and monitor their diet?

RQ2: How would the participants evaluate the usability of the DIMA-P?

RQ3: What kind of behavioral changes would be instigated through the DIMA-P usage?

RQ4: How would the participants’ IDWGs vary over the course of this study?

Even though this DIMA-P study was conducted almost a decade ago, the above questions are still relevant. They underscore the need to address healthcare disparities and promote digital inclusivity by exploring diverse design approaches. Moreover, this study is an example of a theory-based digital health intervention that was aimed at facilitating behavior change and supporting individuals in achieving long-term health goals through self-monitoring. Such studies are still rare. As the healthcare landscape evolves, the lessons from this study serve as a compelling argument for the ongoing development of inclusive, theory-based, and user-centric digital health solutions.

## 2. Background

### 2.1. Overview of Design Studies

The DIMA and DIMA-P were developed through a user-centered design process that was iterative and incremental, undergoing four revisions, three β-tests, and a comprehensive review by health professionals. Outlined below, the design insights derived from this process reflect a deep understanding of the users’ environment, needs, and limitations.

- Match food categories with users’ mental models: Hemodialysis patients think of food items in terms of categories [22]. In particular, they are familiar with USFDA food groups through diet recall sessions with dietitians. They also think of categorizing food items as “what to eat” and “what not to eat”. Moreover, they frequently consume certain items to avoid overshooting their dietary restrictions.
- Incorporate meal building functionality: hemodialysis patients record their meals by recalling items one at a time [22], which allows for a more accurate tracking of their intake.
- Utilize a linear navigation style: The target users prefer to record their meals using a linear guide, which provides a clear, step-by-step pathway through tasks [19]. This approach reduces the cognitive load and makes the application easier to use for patients, especially those who may not be technically savvy.
- Offer real-time and personalized feedback: Healthcare professionals believe that offering immediate feedback on nutrient intake allows patients to make informed dietary choices on the fly [23]. The feedback should be customized to each patient’s dietary needs, displaying crucial information, such as fluid, sodium, and potassium levels.
- Use intuitive icons for feedback: The feedback icons should be designed to be intuitive [23,24]. The icons for restricted nutrients should provide visual cues of approaching limits as they are consumed, while the icons for essential nutrients should indicate the achievement of daily goals.
- Include portion estimation aids: hemodialysis patients with varying literacy skills evaluate the use of non-life-sized icons representing 28 common aids for estimating portion sizes, such as a deck of cards or a golf ball [20,21].
- Conduct a pilot study: We conducted a six-week, in situ evaluation where the target population used the DIMA, enhanced with an interface for estimating fluid portion sizes [18]. Beyond validating the appropriateness of the icons and establishing portion estimation requirements, the feedback from the participants enabled us to identify behavioral health constructs that could help people accurately track and effectively monitor their portion intake. These constructs aligned with those specified by the Integrated Theory of Health Behavior Change (ITHBC) [30], which suggests that health behavior change can be facilitated by fostering knowledge and beliefs, increasing self-regulation skills, and promoting social facilitation.

### 2.2. Theory behind DIMA-P

**Figure 1:**
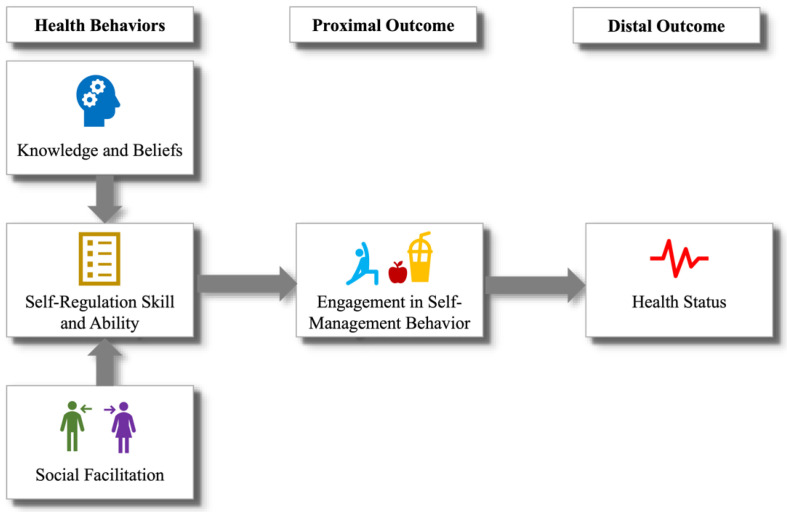
Visual model of the Integrated Theory of Health Behavior Change.

The ITHBC (Figure 1) is a framework that provides a comprehensive understanding of the processes through which interventions may promote behavior change and through which those changes may be maintained over time. According to the ITHBC, three main factors influence health behavior change:

i. Knowledge and belief enhancement: The theory posits that increasing a person’s knowledge about their health condition and related behaviors can positively influence their beliefs about health and illness. This can involve educating individuals about the causes, consequences, and management of their health conditions, thereby fostering a better understanding of and belief in the efficacy of health behaviors.
ii. Self-regulation skills and abilities: ITHBC emphasizes the importance of self-regulation in health behavior change. This includes behavioral aspects such as goal setting, self-monitoring, problem-solving, and decision-making skills. The theory suggests that, by enhancing these skills, individuals are better equipped to initiate and maintain health-promoting behaviors. For example, goal setting might involve defining specific, measurable, achievable, relevant, and time-bound (SMART) objectives for health behavior, while self-monitoring could include keeping a diary of dietary intake or physical activity.
iii. Social facilitation: The model emphasizes the role of social support and interaction in facilitating health behavior change. This includes support from healthcare providers, family, friends, and community. Effective communication, encouragement, and practical assistance from these social networks can significantly influence an individual’s motivation and ability to engage in and sustain health behaviors.

At its core, ITHBC suggests that these constructs lead to sustained engagement in self-management behaviors that are ultimately responsible for improved health outcomes. Such behaviors can range from medication adherence to lifestyle changes like increased physical activity or healthier eating habits.

## 3. Materials and Methods

In this section, we outline the pilot study, where we conducted an in situ usability and usage evaluation of the DIMA-P, examining the changes in participants’ behaviors and IDWGs because of the usage of the DIMA-P.

### 3.1. Intervention Design

**Figure 2:**
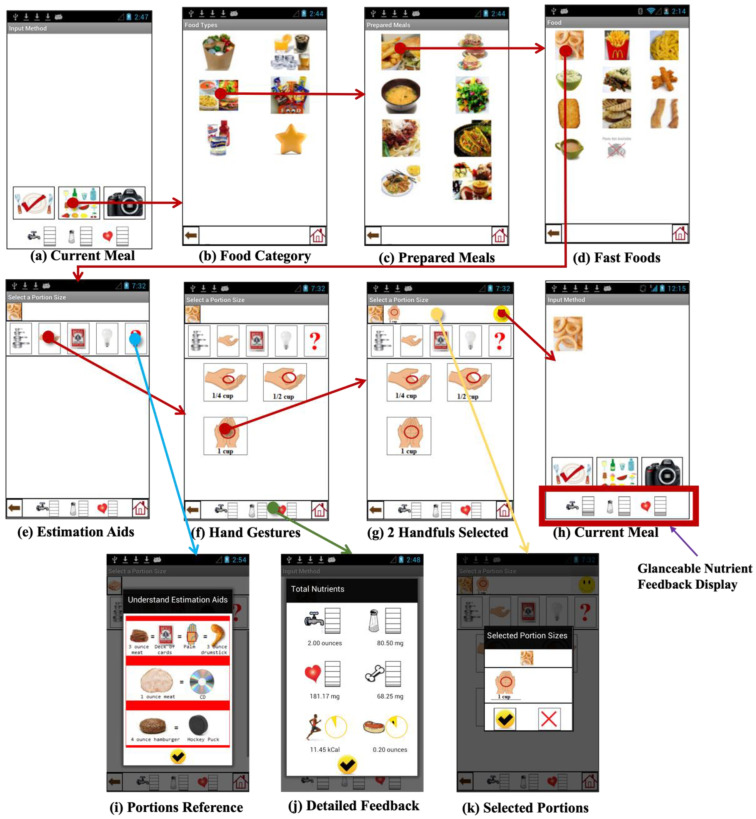
Using the portion estimator to record one cup of grapes and using various constructs of the ITHBC: (**a**) current meal; (**b**) food categories; (**c**) prepared meals; (**d**) fast foods; (**e**) estimation aids; (**f**) hand gestures; (**g**) two handfuls selected; (**h**) current meal items (on main page); (**i**) portions reference; (**j**) detailed feedback; and (**k**) portions review with delete button.

The DIMA-P allows users to record their meals and provides them with the ability to adjust their portion intake by displaying cumulative nutritional values. To log a food item, users first locate it by navigating through a series of pilot-tested DIMA interfaces, following a straightforward linear navigation style. The initial page features two icons related to meal recording: (a) ‘New Meal’ and (b) ‘Saved Meals’. The ‘New Meal’ icon allows the user to record all the food items they have consumed for a meal. Clicking this icon takes the user to the current meal page (Figure 2a), from where they can navigate to the food categories page (Figure 2b), which categorizes foods based on the mental model of the target population. It specifically includes icons for groceries, beverages, prepared dishes, snacks, supplements, and favorites. The ‘Favorites’ category enables patients to quickly log frequently consumed foods. The other categories are strategically designed to limit the number of items on a single screen, thereby facilitating a search for the desired food items. Selecting any of these categories reveals further sub-categories or a food list belonging to that category (Figure 2c). For example, choosing the grocery icon reveals food groups such as fruits, vegetables, meats, dairy, grains, and condiments, whereas choosing the beverage icon reveals different beverages consumed by the users. In sum, the user traverses a set of food category pages to arrive at a page where they can select and record the target food item (Figure 2d).

After selecting an item, the DIMA-P generates a portion estimator interface (PSEI) (Figure 2e), which is based on the food’s shape, size, and physical state at room temperature. The user then selects an appropriate portion size using this interface. The DIMA-P retrieves the food’s nutritional value from its database, adjusts it based on the selected portion size, and displays it via its nutritional feedback feature described in detail below. The nutritional database for the DIMA-P was sourced from the USFDA and includes fluid, sodium, potassium, phosphorus, protein, and caloric values for one serving of approximately 750 different food items. After selecting the portion size, the user is redirected to the current meal page to view the selected item. At this point, the user has several options: (a) save the meal by selecting the check icon, (b) add another item to the meal, or (c) take a photo of the meal. The current meal page also features a glanceable nutritional display that showcases the total intake of fluids, sodium, and potassium. By clicking on the glanceable nutritional display (Figure 2j), the user can access detailed information about the intake of other essential nutrients, such as phosphorus, protein, and calories on the Detailed Feedback page (Figure 2j).

The ‘Saved Meals’ icon on the main page directs the user to a location where all the previously entered meals are stored. Here, the user can find the following information about each previously recorded meal’s chosen food items: estimated portion sizes, the amount of essential nutrients consumed, whether they stayed within the prescribed nutrient limits, and the time and date of the recording. The purpose of this page is to enable self-reflection and self-regulation, which can facilitate behavioral changes.

### 3.2. ITHBC Implementation

The DIMA-P app’s design intricately incorporates the constructs of the ITHBC, enhancing its utility in promoting health behavior change among hemodialysis patients. Here is how each of the ITHBC constructs—knowledge and belief enhancement, self-regulation skills, and social facilitation—are embedded within the application’s functionality:

#### 3.2.1. Knowledge and Belief Enhancement

(a) Nutritional feedback: The DIMA-P provides immediate, visible feedback on the nutritional values of foods as users log their meals. This feedback consists of a glanceable display (Figure 2h), featuring representative icons (e.g., water tap, saltshaker, and heart) for the three critical nutrients that hemodialysis patients need to monitor: fluid, sodium, and potassium [22]. Each icon is linked to a bar chart that fills up as that nutrient is consumed. This helps to educate users about their dietary intake in real time, which enhances their knowledge about how different foods affect their health.
(b) Detailed nutritional displays and feedback pages: By offering detailed information on nutrient intake (Figure 2j) and how it aligns with dietary limits, the app helps reinforce beliefs about the importance of diet management. This ongoing educational component is crucial for modifying users’ dietary habits and beliefs about their health management.
(c) Portions reference: The DIMA-P clarifies how to interpret food portion sizes with the physical sizes of visual aids for specific food groups (Figure 2i). This content was sourced from online educational resources on portion size estimation and verified with a dietitian.

#### 3.2.2. Self-Regulation Skills and Abilities

(a) Portion Estimator Interfaces (PSEI): These interfaces (e.g., Figure 2e,f) allow users to adjust the portion sizes based on visual aids, which helps them to manage their dietary intake more accurately. The ability to adjust portion sizes directly fosters self-regulation skills, as users learn to align their dietary intake with their health goals.
(b) Nutrient displays and portions review: The visual representation of nutrient consumption (Figure 2h,j) (e.g., bar charts that fill up with nutrient consumption) allows users to actively monitor their diet throughout the day. This supports self-monitoring—one of the key self-regulation skills. If a nutrient’s intake exceeds a safe threshold (indicated by the bar turning red), the user can immediately adjust their portions using the portions review feature (Figure 2k), effectively utilizing problem-solving and decision-making skills to manage their diet.
(c) Saved meals: By accessing historical meal data (the checked plate icon in Figure 2a), users can review their dietary patterns over time, fostering reflective learning and better planning for future meals, which enhances their ability to regulate their dietary behavior.

#### 3.2.3. Social Facilitation

While the DIMA-P is primarily a self-use tool, its design indirectly supports social facilitation:

(a) Sharing of dietary data: Users can potentially show their dietary logs or nutritional status to healthcare providers or family members, who can then provide encouragement, advice, or even direct intervention. This sharing process can enhance the social support system, which is crucial for sustained behavioral change.
(b) Symbolic social support [31]: features like compliance indicators and real-time feedback (e.g., icons that react to dietary adherence) can serve as forms of symbolic social support, motivating users through positive reinforcement.

Overall, the DIMA-P’s design leverages the constructs of the ITHBC, integrating them into a comprehensive tool that not only helps users manage their diet through direct interaction but also educates and encourages them to develop long-term healthy eating habits. The app’s functionality in facilitating self-regulation, enhancing knowledge, and indirectly promoting social facilitation makes it a powerful ally in health management for hemodialysis patients, offering a practical application of behavior change theory in a clinical context.

### 3.3. Study Design and Evaluations

After receiving approval from the ethics board, we recruited patients from an urban dialysis facility. The interviews were conducted at the facility during the first two hours of dialysis to accommodate the participants’ comfort and schedules. The eligible participants were those over 18 years old and cognitively able according to the Mini Mental Status Exam (MMSE), a 30-point screening questionnaire that assesses orientation, memory, attention, and executive functions [32]. The individuals who score at or above 26 on the MMSE are classified as cognitively intact or able to maintain a correct train of thought while performing a task. The participants received USD 25 after the baseline interview, another USD 25 after self-monitoring for six weeks, and a final USD 25 after the end-of-study interview. The compensation was not tied to the DIMA-P usage. The main details of the study have been summarized in Table 1.

#### 3.3.1. Baseline Phase

The baseline assessments were completed over two sessions. At the first meeting, the participants completed a background questionnaire. They also took two literacy tests: the Newest Vital Sign (NVS) test and Rapid Estimate of Adult Literacy in Medicine (REALM). The NVS [33] test is designed to assess the ability to read, understand, and apply health information by asking questions based on a nutrition label. The test consists of six questions for 1 point each. A score of 4 and above indicates adequate literacy. The REALM [34] is a medical word recognition and word pronunciation test, consisting of 66 commonly used medical words. The subject is asked to read aloud each word moving downwards in a column. Any word that is not attempted or is mispronounced is counted as an error. A score of less than 61 on the REALM is an indicator of below 9th grade literacy and a need for low-literacy reading materials. A score of 61 or higher indicates a reading level of 9th grade and above, which is an indicator of adequate literacy.

During the second meeting, we trained the participants to use both the mobile phone and the DIMA-P. The training was reinforced with a 25-page manual that visually illustrated tasks such as taking a picture, entering a food item, and using the estimator. The participants had to successfully complete thirteen test tasks to demonstrate their ability to use the DIMA-P independently; otherwise, the training continued into another dialysis session.

After successfully completing the training, the participants performed three Portion Size Estimation Tests (PSETs). These tests assessed their ability to correctly select an image of a portion size from the portion estimation module (Pictures), estimate the sizes of real foods (Apportion), and estimate the volumes of household containers (Containers).

Lastly, we administered the Portion Size Estimation Self-Efficacy Scale (PSESES) [35] and Dietary Self-Regulation Self-Efficacy Scale (DeSReSES)—both modeled on Rossi’s self-efficacy scale [36]. The PSESES aims to measure self-efficacy in estimating portion sizes under various conditions and in using estimation aids and household containers. The purpose of the DeSReSES is to measure an individual’s confidence in managing their diet according to prescribed nutritional guidelines, including tracking food intake, making appropriate dietary choices, adhering to nutritional limits, and applying feedback from health tools or professionals. The questions were read aloud to participants, who then responded to each item on a five-point Likert scale.

#### 3.3.2. Self-Monitoring

After completing the baseline assessment, the participants entered a 6-week-long self-monitoring phase, during which they were expected to record their meals and portion sizes using the DIMA-P. We also asked participants to photograph their meals both before and after eating, using a provided fiducial marker—a deck of cards—to help us estimate food amounts against a standard-sized object during the analysis. The participants’ interactions with the DIMA-P prototype were automatically logged throughout this study. We held face-to-face meetings with the participants during the first two weeks of self-monitoring to resolve issues and answer questions related to this study. For the remainder of this study, the participants could contact us by phone. All the comments and questions that were raised by the participants during our face-to-face and phone interactions were recorded.

#### 3.3.3. End of Self-Monitoring Phase

At the end of six weeks, we conducted the same PSETs and the PSESES and DeSReSES that we had conducted at baseline. Again, all the assessments were completed in the dialysis unit, during the first two hours of the hemodialysis sessions. In addition, we administered custom usability and context-of-use questionnaires to evaluate the usability and use of the DIMA-P over the course of this study. Both questionnaires were specifically developed for an earlier DIMA study, and some items were modified to suit the DIMA-P. We created a custom usability questionnaire based on the goals of this study. Specifically, the questionnaire consisted of 27 items that evaluated user-friendliness, comprehensibility, satisfaction, and usefulness on a 5-point Likert scale (Figures 5–8). The context-of-use (COU) questionnaire consisted of 27 items—3 open and 24 closed—that required binary responses (hard/no and easy/yes) (Figure 9). We also elicited the participants’ perceptions and experiences with the DIMA-P using a semi-structured interviewing technique. Finally, we extracted the interaction logs from the participants’ mobile phones for analysis.

### 3.4. Participants

We approached 15 patients in the dialysis unit who were identified by on-duty nurses as having low literacy and being willing to do this study. Eleven agreed to enroll. Three patients dropped out within the first week—one because of difficulty seeing the screen due to cataracts, and the other two felt too sick to continue. We report on the eight patients who completed this study (Table 2). We recruited two women and six men, all of whom were Black/African American with an average age of 49.4 years (SD = 8.0). All the participants were on Medicaid and lived in low income neighborhoods. On average, the participants had 12.6 years of education (SD = 1.05). One participant (P8†) demonstrated adequate skills in reading, understanding, calculating, and applying written health information according to both the NVS and REALM tests. Two participants (P4* and P7*) performed below adequate levels in both tests. Five (P1, P2, P3, P6, and P9) were either low-literate or low-numerate based on the test results.

Since the participants received the necessary training to use smartphones and the DIMA-P, inexperience with mobile phones was not an exclusion criterion. The participants’ self-reported technology familiarity is classified as: (a) low—used computers at most once a month and had never used a mobile phone before; (b) medium—used computers a few times a month and may have used a mobile phone before; and (c) high—used a computer every day and had also used a mobile phone, including mobile applications. The participants had used computers for chatting, surfing, and social networking. One participant owned a mobile phone at the time of this study, and two had used one previously.

Six participants watched their portion sizes while eating, either by not cooking too much or by eyeballing the amount they put on their plates. Only two participants were familiar with the term ‘estimation aids’. No participant reported tracking or calculating their daily nutritional intake, despite being prescribed strict diets. All the participants were recommended a daily IDWG of 1–1.5 kg by their dietitians.

### 3.5. Analysis

Microsoft Excel’s statistical package was used for all the calculations. For RQ1, we derived the following metrics from the click logs:

(i) Usage frequency, defined as the number of application use days per two weeks of this study;
(ii) Self-monitoring frequency, defined as the average of the daily usage ratings per two weeks of this study. A maximum rating of 2 was assigned to a day with more than one recorded meal, and a minimum rating of 0 was assigned to days with no recorded meals.

For RQ2, we calculated the frequency, mean, and median for the responses to the usability and COU questionnaires [37].

For RQ3, the following data were collected and analyzed:

(a) Behavioral changes were identified using a qualitative analysis of the COU questionnaire and interview responses.
(b) Self-regulation skills were assessed by calculating the change in the Portion Size Estimation Test scores over the course of this study, obtained by subtracting the pre-study test scores from the post-study test scores.
(c) Self-regulation self-efficacy was evaluated by comparing the pre- and post-study DeSReSES scores.

Lastly, for RQ4, we analyzed the participants’ IDWGs collected during this study. Hemodialysis patients weigh themselves both before and after dialysis treatment on the first and last day of the week, and unit nurses log these weights. The IDWG was determined by subtracting the post-dialysis weight from the last dialysis session from the pre-dialysis weight of the current session, divided by the number of intervening days. Approximately, 15% of the IDWG values were missing because patients often forgot to weigh themselves at the end of their treatment. P6’s IDWG values were not available.

## 4. Results

**RQ1: How would participants use the DIMA-P to track and monitor their diet?**

*Usage Pattern.* The data presented in Table 3 reveal varying degrees of participant engagement with the DIMA-P over the course of this study. Five participants could be considered as frequent users (#), using the application for at least 34 or more days, or at least 81% of the time. P1, P4*, and P6 maintained a high level of usage, actively engaging with the application for almost all the study days. P7* and P8† also demonstrated a strong sustained usage by engaging with the application on at least 34 study days.

Three participants (P2, P3, and P9) could be considered as infrequent users (Ø) as they used the application for 26 or fewer days, or at most 62% of the time. These participants showed a moderate level of engagement with the app. P2’s engagement was cut short due to extended hospitalization during this study. P7* showed a substantial decrease in usage in the final week of this study.

*Feature Usage.* The data reveal a considerable variability in the usage of the different features among the participants. P6# exhibited a high total number of clicks across all features, indicating a robust interaction with the application. P4* and P9 were second in demonstrating a very high usage of all features, except “Detailed Feedback”. P2 had a high number of interactions with “Portions Reference”.

The feature “Portions Review” garnered substantial attention from participants, particularly P4*, P6, and P9, who extensively utilized it and its delete button. This underscores the significance users place on the ability to review and potentially adjust their recorded food portions, highlighting its crucial role in portion size estimation.

The high interaction group of “Portions Reference” consisted of P2, P4*, P6, and P9, showing that both frequent and infrequent users also wanted to learn more about and develop skills around portion estimation.

There was relatively little interest in the “Detailed Feedback” feature among all the participants, except P6, compared to other features. However, it is notable that a condensed version of this information was always visible on the main page and PSEI, without requiring any clicks. This might have reduced the need for viewing this page independently.

**Figure 3:**
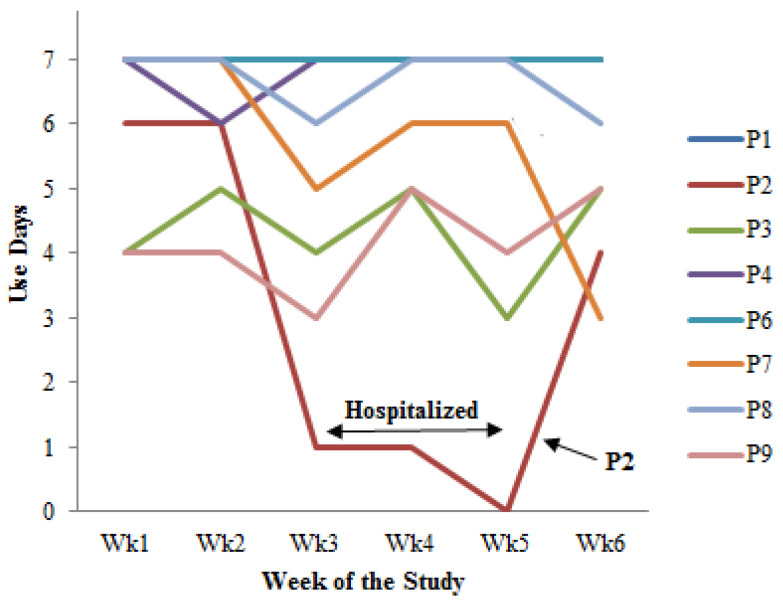
DIMA-P participants’ usage frequency (application use days) by week.

*Usage Frequency.* The usage frequency (Figure 3) was defined as the number of days participants used the application in a week. Most participants did not use the application every day, but they used it frequently and consistently throughout this study, finding a rhythm that met their needs and lifestyle. Four participants (P3, P7*, P8†, and P9) showed a decrease in usage in the third week, coinciding with the cessation of face-to-face meetings with the researcher. Conversely, three participants (including P3 and P9) increased their usage in the last week, including one (P2) who had not used the application from the third to the fifth week (3 weeks) due to hospitalization (Figure 3).

**Figure 4:**
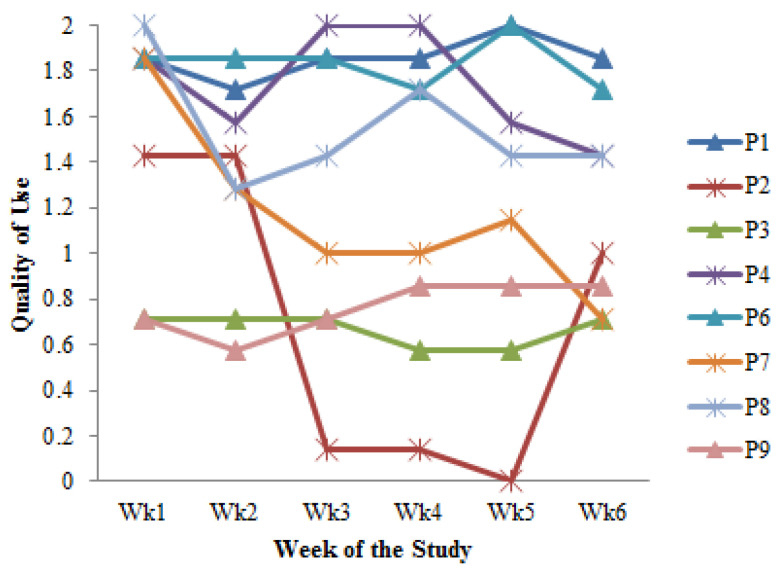
Average weekly quality of use metric indicating self-monitoring frequency by participant.

*Self-Monitoring Frequency.* We developed a quality of use metric where a day was rated High (2) for more than one meal recorded with its portion size, Medium (1) for a single meal recorded with its portion size, and Low (0) for no recording. Four participants consistently maintained a high quality of use (1.5–2.0), two maintained a low quality (0.5–0.9), and two others decreased from a high to a medium quality of use in the final two weeks. Hence, most participants interacted with the DIMA-P multiple times daily throughout this study (Figure 4).

One participant, i.e., P7*, exhibited a significant decrease in quality of use over the course of this study, starting from 1.8 to 1 and 0.6. This suggests that, after the initial excitement of using the app, the participant started to lose interest in recording, a phenomenon referred to as the novelty effect.

A simple linear regression revealed a high correlation between usage and self-monitoring frequencies (correlation coefficient (r) = 0.94). The model also indicated that approximately 89% of the variability in the usage frequency could be explained by a linear relationship with self-monitoring frequency (determination coefficient (r^2^) = 0.89). Moreover, there was a high likelihood that the relationship observed was statistically significant (*p*-value = 0.0014).

**RQ2: How would participants evaluate the usability of the DIMA-P?**

**Figure 5:**
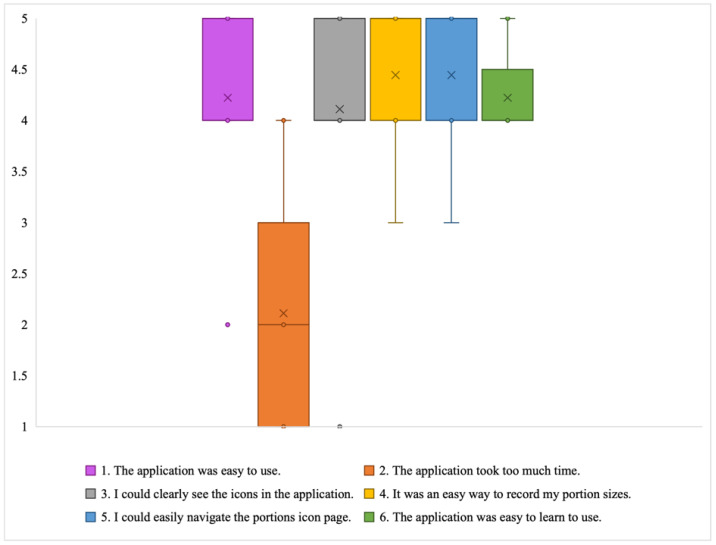
Box plot showing distribution of Likert scale responses for user friendliness subscale (X = mean; and horizontal line = median).

The results from the usability questionnaire are described below. The main results can be described as follows:

*User Friendliness*: The app was well received by the participants in terms of ease of use and the visibility of the icons (Figure 5). The participants agreed that the application provided an easy way to record portion sizes. Most participants were able to use the app without feeling that it was too time-consuming to use (explained in the Time Efficiency Section). The participants thought that navigating the portion size estimation interface to find an appropriate portion icon was easy. Everyone agreed with the statement that the application was easy to learn to use. One participant commented, “I think it was excellent, I could easily find objects and pictures I could relate to on the screen”, (P1).

*Time Efficiency:* On average, the participants spent 43.54 s to record a food item without including portion size, whereas the average time to record a food item along with its portion size was 171.84 s. The Student’s *t*-test confirmed that two recording times were significantly different statistically (*p* < 0.01), i.e., recording food with its portion size was more time-consuming compared to recording the food item alone. The analysis showed that the average recording time did not change over the course of this study, reflecting the inherent complexity of accurately logging portion sizes alongside food items. According to item 2 on the user friendliness scale, two participants agreed with the fact that the app took too much time to record, while the rest were satisfied with the amount of time they spent on the app. However, according to the end-of-study interviews, the participants wanted to use the application regardless of the amount of time it took, because they perceived several long-term benefits, such as helping them lose weight, control their blood phosphorus levels, and increase awareness about their diets. The participants considered these goals important as they are needed to qualify for a kidney transplant; therefore, the extra time and effort required to input the portion sizes were not an issue for many participants.

**Figure 6:**
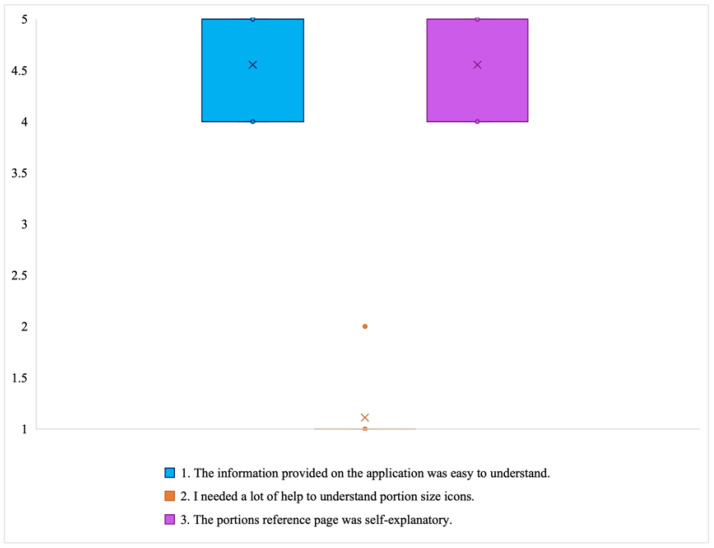
Box plot showing distribution of Likert scale responses for comprehensibility subscale (X = mean; and horizontal line = median).

*Comprehensibility*: The participants’ responses to the comprehensibility subscale are presented in Figure 6. The participants found the information provided by the application to be straightforward and easy to comprehend, as evidenced by a median score of 5. Most users reported being able to understand the portion size icons without much assistance, suggesting that the design was intuitive. “It was enough for me to understand how to record” (P2). They found the portions reference page was self-explanatory. From the interviews, we learned that whenever participants found it challenging to reconcile what they already knew with the information displayed on the screen, they turned to the portions reference page for consultation, which helped them trust the information that was presented in the application: “It is a good reminder because you forget how much a ladle or a serving spoon holds” (P3).

One participant reported that he kept forgetting how to interpret the nutrient icons on the feedback bar: “I did not remember which icon was for which nutrient, I just tried to make sure that nothing was too high (red). I read it as my heart is okay, my bone is okay, run time and meat are okay but not sure how to interpret last two” (P7*). This suggests that there was variability in the participants’ ability to understand the interface, as well as the fact that some parts of the application were more comprehensible than others.

**Figure 7:**
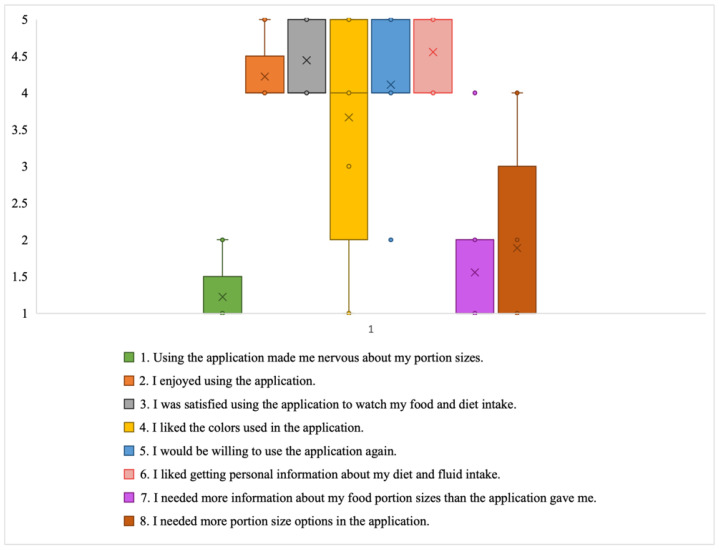
Box plot showing distribution of Likert scale responses for satisfaction subscale (X = mean; and horizontal line = median).

*Satisfaction:* The participants enjoyed their recording experiences, scoring a median of 4 (Figure 7). The participants appreciated receiving personalized feedback, which did not induce nervousness (median ≥ 4). Although there was a range of opinions about the color scheme of the application, most were satisfied with the look and feel of the DIMA-P, with a median satisfaction score of 4. Some thought that the graphics in the application could be made ‘cooler’ looking. While most were satisfied with the available information (median = 4), about one-third of the participants indicated that they would benefit from additional information, such as meal suggestions along with recommended portion sizes. Additionally, two participants expressed a desire to receive estimates of their IDWGs when consuming a food item, in addition to nutritional feedback (satisfaction: item 6).

Although most participants could find their target icons for portion sizes (median = 4), some needed more options. For example, two DIMA-P participants wanted more portion size options for meals served in restaurants, and two wanted additional portion size options or more explanation of how to record slices of fruits. Another participant suggested that the app should be made more dynamic, showing what they should eat at the current time based on allocated nutrient amounts: “People need to know what appropriate portion size is” (P7*). Another participant stated, “Measuring cups were confusing in some places like fruits. Showing a symbol of how much you ate is better than showing a measuring cup. Like hand is better for meat, salad, and snacks” (P6).

**Figure 8:**
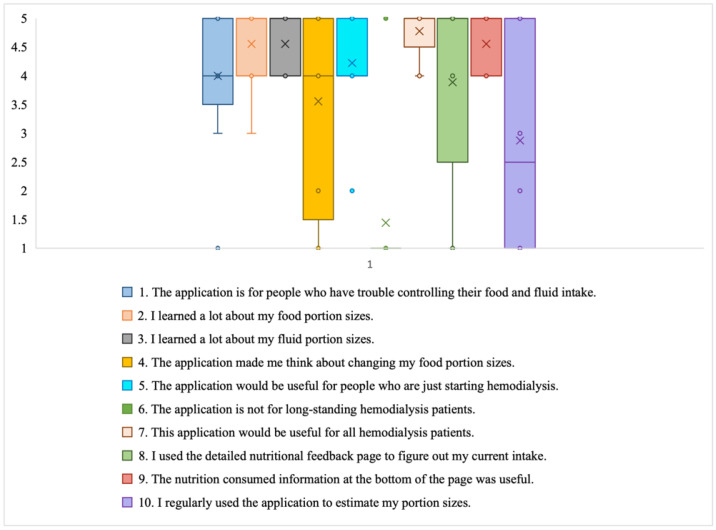
Box plot showing distribution of Likert scale responses for usefulness subscale. (X = Mean; and h Horizontal line = Median).

*Usefulness:* The participants believed that the DIMA-P would be useful (Figure 8) for all dialysis patients, with a median score of at least 4. They also thought the application would be beneficial for those having difficulty controlling their dietary intake (median ≥ 4). The participants acknowledged that the application helped them gain insights into their diet (median = 4), fluid consumption (median = 4), and portion sizes (median = 5).

All participants found the glanceable nutrient display at the bottom of the pages of the DIMA-P to be useful for self-monitoring, with a median score of at least 4. However, not all the participants used the detailed nutritional feedback page, matching the results from the click logs analysis. This indicates that the participants preferred to keep track of their main nutrients by visualizing the readily viewable icons. One participant explained, “I was looking at the cup at the bottom filling up and going red, then I know I can’t eat that right now” (P4*).

Regarding items 4 and 10, the participants explained that they did not regularly use the application to estimate their portion sizes. This is because many participants had been on dialysis for a long time, and they were already consuming the amounts that helped them stay healthy: “I have been on dialysis for some time now, so I know my measurements. I used it to make sure I was not too high on certain things. If something was too high, I would cut down” (P7*).

Many used the app to increase their knowledge and change their behaviors, rather than relying on the app all the time. However, everyone agreed that regularly using the app to document their meals was important for staying aware of their food consumption and making better dietary choices for throughout the day: “It made me realize that when I had that food, I do not have that much of that food” (P8†).

**RQ3: What kind of behavioral changes would be instigated through the DIMA-P usage?**

**Figure 9:**
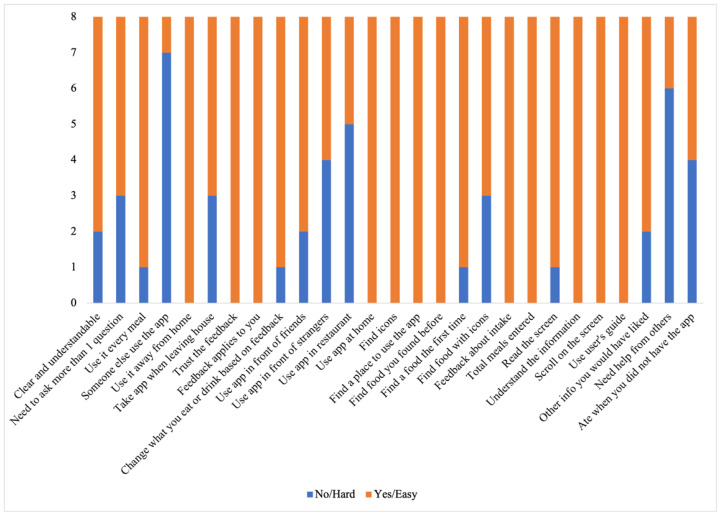
Response distribution of the context-of-use questionnaire.

*Behavioral Changes:* Based on the COU questionnaire (Figure 9) and interview responses, we gained the following insights into the behavioral changes that may have been instigated using the DIMA-P. These changes were primarily related to the use of and interaction with the application, as well as dietary behavior changes. Here are some observations based on the COU responses and interview analysis:

- Cultivating a meal tracking habit: The participants unanimously acknowledged the utility of using the DIMA-P for meal tracking, and, as indicated by the context-of-use (COU) questionnaires, they reported using it consistently with each meal, whether at home or away from home. However, a more detailed examination of their click log data (Figure 3) revealed that not all the participants recorded their meals every single day throughout this study. Additionally, some participants mentioned in the interviews that they occasionally deferred meal recording and inputted their dietary information retrospectively. For instance, one participant explained, “There were days when I added everything at the end of the day” (P3).
  Furthermore, the participants disclosed variations in their daily eating patterns. Some individuals revealed that they consumed only one meal a day or occasionally skipped meals, especially on dialysis days when they were not feeling well. As one participant noted, “When I am not feeling too good especially on dialysis days, I tend to eat less” (P9). Consequently, the lower frequency of use or quality metrics should not be interpreted as a lack of meal tracking; rather, it reflects the flexible and adaptive nature of how the participants engaged with the application to suit their unique dietary routines and health conditions.
- Increased interest in self-monitoring: The users reported that they were easily able to find all the meals they had entered into the application, which supports the self-monitoring aspect of behavior change. The participants further explained that the app helped them track and review their meals, helping them decide what to eat later: “Documenting helps you make better choices and how much to eat” (P3).
- Using the application as a problem-solving tool: The participants, particularly those who were infrequent users, tended to use the application as a problem-solving tool rather than for tracking every meal. Many of these participants reported that they did not vary their diet much (typically, consuming items they had experimented with before). They preferred to stick with familiar, safe choices. As a result, using the app daily did not seem necessary to them. Instead, they primarily relied on the app to make informed decisions about unfamiliar foods or those they did not consume regularly. They often used the app’s reference feature to determine the correct portion sizes or experimented by trying out different meal options as exemplified by P3, “I’m pretty consistent with my diet, so I didn’t use the app every day. I kept it handy for those times when I needed to confirm if something was okay to eat”.
- Adherence to application feedback: All the participants reported changing what they were eating and drinking based on the feedback from the application, indicating that the DIMA-P was effective in helping the participants adhere to their dietary restrictions: “I slowed down when it *[nutrient feedback icon]* was red and I tried to watch what I was eating” (P2).
- Improvement in nutritional intake: Several participants highlighted the positive impact of the app on their dietary habits. One participant expressed, “It made me realize I was not eating enough protein, so I started incorporating more protein into my diet” (P7*). This observation suggests that the application not only facilitated portion size estimation but also raised awareness about nutritional aspects, encouraging users to make healthier dietary choices. One participant also reported that the app helped him lose weight. “I lost 3 kg; it helped me see how much I was eating. It will help me even more after that I am done losing more weight” (P8†).
- Learning to trust the application: Some participants stated that initially they had a hard time believing some of the information presented in the application, such as some portion size images being hard to believe. These participants engaged in experimentation, which ultimately led them to believe the application and correct their own misperceptions: “Some of the portion sizes are not believable, but when you look at the actual size of the object, it becomes believable” (P2). In particular, according to the end-of-study COU questionnaires, most of the participants stated that they trusted the nutritional information in the DIMA-P. This is important for users to act on the information and advice provided.

*Self-Regulation Ability.* The results of the PSETs were used to test the participants’ ability to estimate portion sizes, which indirectly provides evidence of their ability to self-regulate (Table 4). In the first test, i.e., Pictures, all the participants, regardless of their literacy and technical skills and their frequency of use, had higher post-study scores. This suggests that after using the DIMA-P, the participants were able to improve their ability to select accurate visual estimates of given portion sizes. Interestingly, the improvement was observed across participants with varying literacy levels and technical skills, indicating that the app’s design was accessible and intuitive enough to bridge potential knowledge gaps.

In the Measure test, four participants had higher, one the same, and three lower post-study scores. The improvements and non-improvements were observed across all demographics, suggesting that the participants’ ability to measure portion sizes was not strongly correlated with their literacy levels, technical skills, or usage frequencies.

Finally, four participants scored higher and four scored the same on the post-study Containers test. Again, we observed improvements and no changes across all demographics, regardless of the participants’ literacy levels, technical skills, and usage frequencies. The mixed results may highlight the need for more tailored interventions or additional training to better support participants in accurately measuring food portions, particularly for those with lower literacy or technical skills.

**Figure 10:**
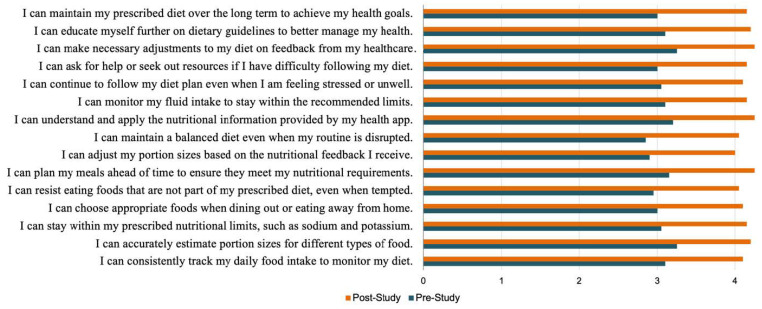
Mean pre- and post-study diet self-regulation self-efficacy scores.

*Dietary Self-Regulation Self-Efficacy.* The mean scores on the DeSReSES questionnaire improved post-study on all items (Figure 10). These results indicate that the participants gained greater confidence in their ability to regulate their diet and adhere to prescribed nutritional limits as a result of using the DIMA-P.

The participants’ post-study comments further support these results. For instance, one participant mentioned that the application helped them practice what was preached to them by their providers, “I knew about estimation aids from my dietitian, but I never used them. This tool helped me practice what my dietitian always tells me to do and improve my intake” (P1). Another concept that emerged was the participants’ confidence in their understanding of their own dietary practices. One participant stated, “I was already measuring my fluid, but it helped me realize how quickly I ate 4 ounces of peanut butter” (P3). Another attributed their increase in confidence to the actionable knowledge disseminated by the application, “It added a lot of knowledge. It showed me what the heck I was doing and how to do it better” (P7*).

**RQ4: How would the participants’ interdialytic weight gains vary over the course of this study?**

**Figure 11:**
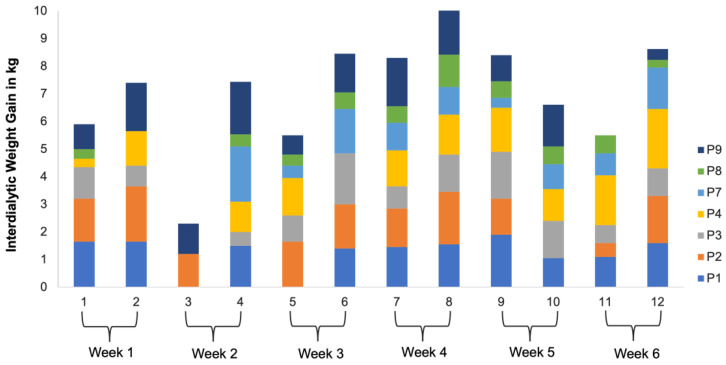
Individual interdialytic weight gains over the course of this study.

**Figure 12:**
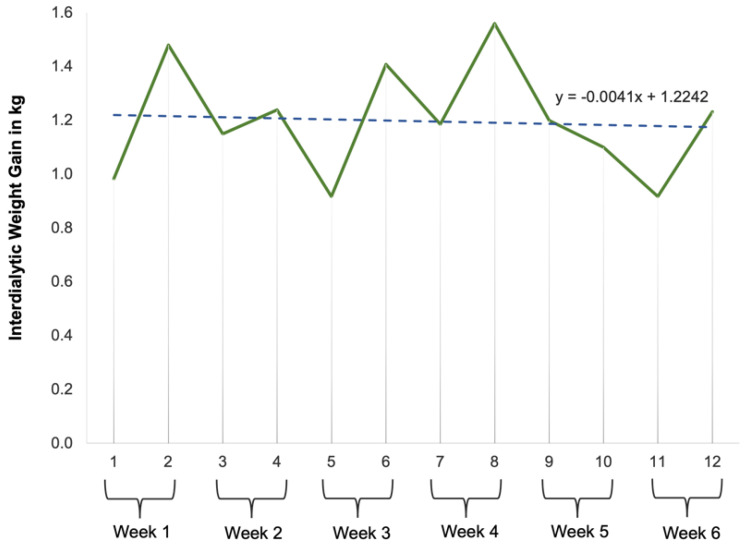
Mean interdialytic weight gain across this study (dotted line indicates the trend).

The participants’ actual weights were captured at each dialysis session over the course of this study (Figure 11). To obtain the interdialytic weight gain (IDWG), the weights from two consecutive dialysis sessions were subtracted and divided by the number of intervening days. For example, if a participant’s weight was 70 kg on Monday and 72 kg on Wednesday, the IDWG would be calculated as (72–70 kg)/2 days = 1 kg/day. The average IDWGs of all the participants fluctuated across this study, oscillating above and below the 1.0 kg mark, the ideal and recommended IDWG for dialysis patients, but within the range of 0.8 kg to 1.6 kg (Figure 12). The linear equation (y = −0.0041x + 1.2242) for the chart showing the mean IDWG implies a slight negative trend. The trendline (dotted line in Figure 12) begins just above 1.2242, predicts a slight downward trajectory of the rate of 0.00041 kg per session, and approaches a value just below 1 by the final session. This indicates that the overall mean IDWG decreased marginally as this study progressed. This suggests a slight variability in IDWG over the course of this study. Overall, these findings indicate that during the DIMA-P use, the participants were able to maintain their IDWGs at the recommended level of 1.0 kg.

## 5. Discussion

### 5.1. Principal Findings

Given that mobile health applications are typically challenging to use by people with limited literacy skills [38], the focus of this research was to assess the usability and feasibility of a mobile health application with a chronically ill, low-literacy population. The app was designed with feedback from the target population using an extensive user-centered design process. In this study, we recruited eight participants with varying literacy and numeracy skills. Seven participants were low-literacy or low-numeracy or both, while one participant had adequate literacy and numeracy. This study yielded insightful findings regarding the engagement and interaction of this population with a theory-informed dietary intake monitoring application.

The user engagement patterns revealed that most participants did not use the app daily but adapted its use to fit their personal schedules and needs. Five participants emerged as frequent users; this included two participants who had both low literacy and low numeracy skills. This indicates that the app was user-friendly, and the participants easily integrated it into their daily routines. The remaining three participants were infrequent users, using the application for fewer than 26 (out of 42) study days. One participant’s lower engagement was due to hospitalization, while the others indicated that they either did not eat every day or did not need the application daily because they were already aware of what they were eating. There was also a significant decline in app usage among infrequent users when face-to-face interactions with researchers were not available, suggesting that direct support played a role in motivating consistent app usage in some users.

The observed variable usage pattern demonstrates the impact of external and internal factors such as user goals, illness, eating patterns, etc., on app usage. Future research should explore these aspects in this population in greater detail to further enhance the usability and usefulness of diet tracking applications. For instance, while the DIMA-P was primarily designed to encourage the participants to record their meals and receive feedback, many users also utilized it to make decisions about what to eat by experimenting with different meal choices. This behavior exemplifies technology appropriation, where users adapt the app’s intended purpose to meet their own needs. Investigating the other appropriated uses could help ensure that the app includes all the necessary features to support users in achieving their goals more effectively.

In terms of usability, the participants were satisfied with the application’s ease of use and the clarity of its portion size icons. This suggests that our icon-based user interface design strategy made the application accessible to users with both limited literacy and technological skills. However, a few icons were initially difficult for participants to interpret, and many needed additional icons that better reflected their personal dietary intake. These challenges highlight the difficulty of designing iconography that is both intuitive and universally applicable, while accommodating the diverse dietary habits and preferences of users.

Advanced technologies like DALL-E [39] can produce highly accurate and diverse images. When combined with prompt engineering techniques, these AI-driven visuals could be tailored to create more personalized, culturally relevant, and easily understandable icons that resonate with users. The design of such systems should prioritize automating the prompt engineering process as much as possible, minimizing the need for user input to ensure accessibility regardless of users’ literacy skills. Instead of requiring users to generate or modify prompts themselves, the system could offer pre-configured options or simple, guided interactions (e.g., multimodal options) that allow users to select or adjust the visuals based on their preferences or needs. This approach would ensure that even those with limited literacy skills could benefit from tailored, AI-driven visuals without the complexity of direct prompt engineering.

Even though tracking both dietary intake and portion sizes took longer than recording a meal without its portion size, the participants did not think that this extra time deterred them from using the application. They recognized the long-term benefits of the app, such as aiding in weight management and dietary awareness—critical factors for patients needing strict diet adherence. This finding validates the fact that the participants found the app to be a valuable tool for monitoring and tracking dietary intake, as well as learning about dietary intake.

While the app does meet its intended purpose with the current design, its usefulness can be enhanced by further considering the participants’ suggestions. For example, the participants expressed a desire for real-time, personalized dietary suggestions (i.e., what to eat and how much to eat) and a more transparent linkage between dietary intake and health outcomes, i.e., how a certain dietary item would impact their weight gain between dialysis sessions. Machine learning and real-time analytics could help bridge the gap between person-specific daily dietary habits and tangible health repercussions, as well as recommend food items to meet a patient’s needs and preferences. However, integrating these features could present several challenges.

Developing machine learning models that provide personalized dietary suggestions and predict health outcomes is quite challenging, as these models must be trained on large datasets and adapt to the unique characteristics of each patient while accounting for various influencing factors [40]. Ensuring the accuracy and reliability of these predictions is crucial in healthcare, since inaccurate suggestions could lead to harmful outcomes. Additionally, patient acceptance and engagement are vital; the technology must be user-friendly and patients need to trust and understand the system. The resource intensity of developing and maintaining such systems adds to the difficulty, as does the need for interdisciplinary collaboration among healthcare professionals, data scientists, and software developers [41]. This highlights the complexity of integrating advanced technological features into self-monitoring tools, necessitating careful consideration, significant investment, and ongoing refinement to ensure their effectiveness and safety. Nonetheless, future research should explore the possibility of developing personalized machine learning models to help people make better dietary choices and develop a better understanding of their health.

### 5.2. Theoretical Contributions

One of the goals of this study was to use the ITHBC framework in the design and functionality of a mobile health application to promote healthy behavior in a chronically ill population. The results show that the app facilitated important behavioral changes (making healthy dietary choices) by enhancing users’ knowledge, supporting the development of self-regulation skills, and integrating into their social environments. Improvements in dietary habits and an increase in self-efficacy regarding portion control were reported, reflecting the app’s effectiveness in enhancing user competence and confidence in managing their diet. This study also monitored the participants’ IDWGs, finding that while there were minor fluctuations, the levels generally stayed within the desired range. This stability in the IDWGs is a positive indication that the DIMA-P was effective in helping participants manage their fluid and dietary intake adequately. This study’s findings contribute to a deeper theoretical understanding of how health behavior interventions can be designed to initiate and sustain change. Below, we discuss the mechanisms by which the design of each ITHBC construct facilitated the dietary behavior.

#### 5.2.1. Knowledge and Beliefs

The application provided real-time feedback on nutritional content and appropriate portion sizes, actively engaging users in understanding the consequences of their dietary choices. The real-time nutritional feedback translated abstract nutritional concepts into concrete visual concepts, bringing knowledge to the fingertips of the users at a crucial moment of decision-making. The application also contained a reference page that provided the participants with information on how to estimate the portions of specific food items using estimation aids. This feature allowed the participants to translate abstract concepts into actionable knowledge, enabling them to accurately portion out specific amounts of food. Hence, the application provided knowledge to bridge the gap between theoretical understanding and real-world application, empowering users to make more informed dietary choices.

The participants were expected to develop the belief that they could effectively manage their dietary intake by accurately estimating portion sizes and understanding the nutritional content of their meals. The real-time feedback and reference page were designed to instill this confidence and reinforce the belief that they have control over their nutritional health and can take proactive steps to improve their health outcomes through better dietary practices.

In terms of knowledge improvement, the data revealed that the participants generally enhanced their ability to visually estimate portion sizes after using the DIMA-P, a skill specifically promoted by the app. However, the results for the measuring and container estimations were mixed, indicating that while some participants improved, this was not consistent across the board. A moderate positive correlation between these skills and literacy levels suggests that literacy may influence the effectiveness of portion size estimation. This finding indicates that future versions of the DIMA-P could benefit from incorporating more comprehensive guidance on alternative methods of portion estimation, thereby helping participants improve their dietary intake behavior more broadly.

The improved scores on the portion estimation skills tests, portion estimation self-efficacy scale, and diet self-regulation scale provide empirical support for the effectiveness of the DIMA-P. These outcomes suggest that the knowledge dissemination features of the app enhanced users’ understanding and beliefs about their dietary behaviors. Additionally, the high comprehensibility ratings of the app indicate that the information was not only accessible but also easily integrated into the users’ belief systems, further supporting their dietary decision-making processes. By grounding the design of the DIMA-P in the ITHBC, the app exemplifies how health behavior interventions can be structured to initiate and sustain dietary behavior change by focusing on the critical role of knowledge and beliefs in shaping and reinforcing healthy behaviors.

#### 5.2.2. Self-Regulation Skills and Abilities

Self-regulation is the process by which individuals control their thoughts, emotions, and behaviors to achieve specific goals [42]. In the context of health and dietary habits, self-regulation is crucial for maintaining healthy behaviors, making informed decisions, and achieving long-term health outcomes. It involves setting goals, monitoring progress, and adjusting behaviors in response to feedback.

The results indicate that the participants used the DIMA-P to record and modify their meal choices (decision-making) to improve their nutritional intake. Each participant established their own usage pattern, characterized by adjusting and self-monitoring their diets (flexibility and commitment), suggesting that they developed self-regulation skills over the course of this study. The presented analysis suggests that three key features of the DIMA-P were instrumental in promoting these self-regulation skills. First, the app provided real-time feedback on nutritional content and portion sizes, enabling the participants to assess whether they were meeting their dietary goals and to make necessary adjustments. Second, positive or negative reinforcement messages helped the participants manage their emotions and thoughts about their food choices, further supporting their ability to regulate their eating behaviors. Lastly, the app’s intuitive and adaptable interface made it easy for the participants to experiment with different meal choices, allowing them to add items, review feedback, and then delete or modify food items as needed. These features collectively supported the participants in developing and sustaining effective self-regulation strategies for healthier eating habits.

The self-regulation skills developed by the participants ultimately led to better dietary management, as evidenced by their relatively stable IDWG levels, which remained within the desired range throughout this study. This stability in the IDWG indicates that the participants were effectively self-regulating their fluid and dietary intake. Moreover, the participants demonstrated flexibility, commitment, problem-solving skills, and consistency in their use of the DIMA-P—critical elements of effective self-regulation [43]. These skills are essential for maintaining long-term behavior change and achieving health goals.

#### 5.2.3. Social Facilitation

While the use of the DIMA-P in social settings, such as restaurants, was less frequent, the application was still employed in these contexts. This suggests that the DIMA-P supported the social facilitation aspect of the ITHBC, as it did not disrupt the participants’ social interactions, and they felt comfortable using it in front of friends. In fact, several participants viewed the app as a status symbol in social settings, indicating that it played a significant role in boosting their self-confidence in dietary monitoring in these situations. Additionally, the participants were comfortable using the app at home in the presence of family members, and many shared it with their caregivers to help achieve their dietary goals (e.g., assisting in meal preparation). However, we recognize that the app could have further enhanced social interactions between the patient and their social networks. For example, it could have included features that allowed for more direct engagement (e.g., ref. [15] supported the crowdsourcing of nutritional intake) or the sharing of tracked meals with social networks, thereby fostering a greater sense of community and support around dietary monitoring. Future research could explore the impact of incorporating these social features on user engagement, motivation, and the sustainability of healthy eating behaviors over time.

Hence, the design of the DIMA-P was effective in aligning with the ITHBC’s core constructs of fostering knowledge, promoting self-regulation, and encouraging social facilitation. The application’s adaptability to users’ individual circumstances further demonstrates its ability to accommodate diverse user needs, ultimately leading to sustained behavior change and improved health outcomes.

## 6. Limitations

While we cannot draw general conclusions about health outcomes due to the small sample size and limited duration of this study, we did observe a variety of behaviors within the study sample that can provide valuable insights. These behaviors include differences in how the participants engaged with the app, adapted it to their individual routines, and changed behaviors based on its feedback. The diversity in these responses highlights the importance of personalized approaches in digital health interventions and suggests areas for further research to better understand how such tools can be optimized to support sustained behavior change across different populations. Moreover, we focused on usability and feasibility assessments of the application. In this regard, both the size and length of our study were suitable for this study [44].

## 7. Conclusions and Future Work

Hemodialysis patients with varying literacy levels could use an icon-based portion size estimation interface. Moreover, they appreciated the accurate estimates of their total nutrient intake, even though the process took a considerable amount of time. The DIMA-P’s design, based on five years of user-centered deign research, in terms of comprehensibility, satisfaction, and usefulness aligned well with users’ needs. The participants recognized its utility, especially for self-monitoring and long-term health management goals like weight loss and transplant eligibility.

Regarding usage frequency, while some participants’ app usage decreased temporarily, possibly due to the novelty effect or external factors, like hospital admission, others increased their usage over time. A strong correlation between usage and self-monitoring frequencies was found, indicating that those who used the application on more weekdays also used it more often each day for self-monitoring purposes.

In terms of outcomes, IDWGs displayed minor fluctuations within a narrow range and a marginal downward trend over time, suggesting a slight but consistent improvement in managing fluid and dietary intakes during the study period.

Overall, the DIMA-P had a multifaceted impact on users, influencing their dietary behaviors, learning, and engagement with self-monitoring practices. The data also indicated that there were improvements in the portion size estimation skills promoted by the application and portion estimation self-efficacy. However, there was also a desire for more varied and diverse portion size images and concrete linkages between intake and outcomes. Such engagement bodes well for sustained personal nutrition management and points towards avenues for further research, such as the use of machine language to support decisions related to portion size intake.

Future iterations of the app and similar interventions could further capitalize on these findings. It could aim to maximize the impact of each ITHBC construct on the user’s journey toward healthier behaviors. Research should refine the mechanisms by which technology can further enhance each component of the ITHBC framework.

We have outlined many future research possibilities throughout the discussion section. Other areas of future research include exploring the longitudinal effects of such interventions. Studies could also focus on adapting this application for different medical conditions and demographics. As mentioned earlier, another avenue for future research involves collaborations among data scientists, human–computer interaction experts, and healthcare professionals for developing artificially intelligent, user-centered, and clinically effective solutions. These collective efforts will not only cater to a diverse user base but also set the stage for groundbreaking advancements in personalized medicine.

## Figures and Tables

**Table 1:** Additional details of the DIMA-P study.

**Total Participants**	8
**Duration**	6 weeks of self-monitoring
**Time**	1 July 2013–30 September 2013
**Contact with Researcher**	First two weeks = face-to-face Last four weeks = via phone
**Baseline Assessments**	Demographics, MMSE, REALM, NVS, PSESES, PSETs, DeSReSES, IDWG, and nutrition portion size estimation self-efficacy
**Self-Monitoring Data**	Meal photographs, IDWG, nutrient intake data (DIMA-P), food items and portion sizes (DIMA-P), DIMA-P click logs, and field notes
**End of Self-Monitoring Assessments**	PSESES, PSETs, DeSReSES, custom usability questionnaire, context-of-use (COU) questionnaire, IDWG, and exit interview

**Table 2:** DIMA-P literacy and technology familiarity levels (* indicates both low literacy and low numeracy; † indicates adequate literacy and numeracy; and the rest have either low literacy or low numeracy).

Participant	Education Years	NVS (Literacy Level)	REALM (Literacy Level)	Tech Familiarity
P1	14	3 (possibly low)	65 (High school)	Medium
P2	13	4 (adequate)	59 (7th–8th grade)	Low
P3	14	2 (possibly low)	66 (High school)	Low
P4 *	12	3 (possibly low)	56 (7th–8th grade)	Low
P6	12	5 (adequate)	47 (7th–8th grade)	Medium
P7 *	13.5	1 (likely low)	45 (7th–8th grade)	Medium
P8 †	12	5 (adequate)	65 (High school)	High
P9	11	5 (adequate)	60 (7th–8th grade)	Medium

**Table 3:** Feature and app usage details (# = frequent users; Ø = infrequent users; * = low literacy + low numeracy; and † = adequate literacy and numeracy).

Participant	Usage Group	Total Use Days (Study Days = 42)	Mean Quality Metric (SD)	Total Clicks (Over 6 Weeks)		
				Portions Reference	Detailed Feedback	Portions Review (Delete)
P1	#	42	1.82 (0.10)	1	4	2 (0)
P2	Ø	18	0.70 (0.64)	12	3	4 (0)
P3	Ø	26	0.67 (0.05)	0	4	1 (0)
P4 *	#	41	0.65 (0.05)	31	3	32 (8)
P6	#	42	1.77 (0.15)	68	13	32 (15)
P7 *	#	34	1.15 (0.37)	3	1	4 (0)
P8 †	#	38	1.52 (0.29)	2	1	5 (0)
P9	Ø	25	0.78 (0.13)	13	2	27 (11)

**Table 4:** Pre- and post-study scores on the three Portion Size Estimation Tests (PSETs). * = low literacy + low numeracy; and † = adequate literacy and numeracy.

Participant	Pictures (Out of 6)		Measures (Out of 10)		Containers (Out of 6)	
	Pre	Post	Pre	Post	Pre	Post
P1	3	6	5	6	4	6
P2	2	4	6	9	2	2
P3	2	4	6	9	2	2
P4 *	3	6	6	4	3	6
P6	6	6	5	6	5	5
P7 *	4	5	4	4	4	4
P8 †	2	6	6	5	2	4
P9	2	5	6	5	1	3

## Data Availability

Data sets generated during the reported study are available from the corresponding author on reasonable request.
